# Biomonitoring of airborne trace elements using *in situ* and transplanted lichens around a recycling industry

**DOI:** 10.1007/s10661-026-15617-2

**Published:** 2026-06-30

**Authors:** Rubén Pérez-González, Violeta Rangel-Osornio, Sonia Trobajo, Estrella Alfaro-Saiz, Ana Belén Fernández-Salegui

**Affiliations:** 1 Department of Biodiversity and Environmental Management (Botany), University of León, 24071 León, Spain; 2 EBUM Herbarium, Universidad Michoacana de San Nicolás de Hidalgo, 58341 Morelia, México

**Keywords:** Atmospheric pollution, Bioaccumulation, Enrichment factor, Distance gradient, *Evernia prunastri*, Potentially toxic elements

## Abstract

The concentrations of six metals (Al, Cr, Cu, Fe, Pb and Zn) were analysed in the lichen *Evernia prunastri* to evaluate the air quality in the surroundings of a tyre and Cu cable recycling industry in Leon, Spain. We evaluated the effect of atmospheric emissions from this industry on lichen samples collected in the area (*in situ*) and on transplants taken from a reference site. The study area was divided into eight transects according to orientation and the sites were located at increasing distance from the pollution source. Pollution maps with Kriging interpolation method were elaborated. Moreover, Generalised Linear Model, Principal Components Analysis and Spearman’s correlations were used. The results showed that the lichen samples collected and exposed near the industry accumulated higher concentrations of these metals, decreasing with the distance and in the direction of the prevailing winds. *In situ* samples showed higher concentration than transplants. Among elements, Cu was significantly higher around the industrial area, followed by Zn. Elements with similar behaviour and common sources were detected. This study highlights the presence of anthropogenic atmospheric pollution associated with the recycling industry, which may pose a potential environmental and public health concern for nearby populations.

## Introduction

Monitoring atmospheric pollution of anthropogenic origin is a highly complex issue due to the large number of potentially hazardous substances involved, the difficulty of estimating their synergistic effects, the substantial spatial and temporal variability of pollution phenomena, and the high cost of sampling instruments (Brunialti & Frati, 2007; Nimis et al., 2000). Moreover, immission measurements require long-term studies with a high number of sampling intervals (Wolterbeek, 2002). Biological monitoring is an effective approach to identify major pollution sources and areas of greatest risk to both the environment and human health, as well as being a quick and inexpensive method (Bargagli et al., 2002). In such cases, biomonitoring proves to be highly useful in assessing atmospheric pollution (Brunialti & Frati, 2007; Chaparro, 2021).

Lichens and mosses are organisms widely used as biomonitors, particularly lichens, for which a vast amount of literature is available (Anderson et al., 2022; Brunialti & Frati, 2007; Chaparro, 2021; Conti & Cecchetti, 2001; Conti et al., 2004; Frati & Brunialti, 2023; Giordani et al., 2019; Hawksworth et al., 2005; Nimis et al., 2000; Pradhan et al., 2025; Sulaiman et al., 2018; Trobajo et al., 2022, 2025). Lichens are the result of a symbiotic association between a fungus (mycobiont) and an algal and/or a cyanobacteria (photobiont) (Anderson et al., 2022). Lichen metabolism depends on the mineral uptake from the atmosphere, they grow very slowly, have no stomata or cuticle to regulate air exchange and accumulate pollutants over the entire surface (see Hawksworth & Grube, 2020 for a more complete definition); therefore, these organisms are highly effective in trapping trace elements from the surrounding environment (Paoli et al., 2015). The concentrations found in their thalli can be correlated with those present in the environment (Conti et al., 2004; Kularatne & De Freitas, 2013; Paoli et al., 2015). *Evernia prunastri* (L.) Ach. is a one of the most used lichen species in bioaccumulation studies (Mallen-Cooper & Cornwell, 2020), due to its easy identification and handling, as well as its cosmopolitan distribution (Bergamaschi et al., 2007; Conti et al., 2004; Loppi & Paoli, 2015; Nannoni et al., 2015; Nimis, 2016; Paoli et al., 2015).

Two possible bioaccumulation techniques can be employed: lichen samples collected *in situ* or transplanted samples. The first technique involves collecting native lichen samples from the study area, which provides information on a variable period of exposure to pollutants. Specifically, the analysis of the complete lichen thallus offers data on prolonged exposure to trace elements (Brunialti & Frati, 2014; Chaparro, 2021). The second technique is used when high levels of pollution have eliminated lichens from the area (lichen desert). In transplant technique, lichen thalli are collected from a clean area and subsequently exposed for a period in the study area to examine the elements accumulated in the lichen thallus (Brunialti & Frati, 2014; Godinho et al., 2009; Trubina et al., 2014). The exposure duration for transplants varies depending on the study, ranging from 15 days to 12 months. However, in most cases, samples are exposed for 1 to 3 months, as transplanted lichens may lose biomass or become saturated with elements, significantly altering their surface structure and physiological performance (Brunialti & Frati, 2014; Giordani et al., 2019; Mikhailova, 2002).

Anthropogenic pollution from the increase in solid waste is generated by economic growth and rising living standards (Tian et al., 2012). Solid waste shredding plays a crucial role in recycling by reducing the size and shape of waste to facilitate its further separation into different materials (Flizikowski et al., 2021). This process is used for mechanical recycling of cables and car tyres (Flizikowski et al., 2021; Yang et al., 2011). An estimated 1 billion tyres are discarded annually, posing a severe environmental and public health risk (Formela, 2021). Tyre accumulation outdoors is a potential risk of uncontrolled combustion (Formela, 2021). For example, in León (Spain), a fire broke out at a recycling industry specialising in tyres and metal recycling. The fire consumed approximately 12,000 tonnes of tyres and took about 22 days to extinguish (Cubillas & Caramazana, 2015).

The burning of tyre rubber can release harmful compounds into the atmosphere, such as sulphur oxides, polycyclic aromatic hydrocarbons, fine particulate matter and other persistent hazardous substances (Formela, 2021). Similarly, the accumulation and shredding of tyres releases pollutants such as potentially toxic elements (PTEs), which can be absorbed by living organisms increasing the risk of respiratory system diseases and cancer (Formela, 2021). The long-term impact of these pollutants on ecosystems causes changes in species composition, structure, and productivity, as well as the presence of PTEs in soil and plants. Numerous studies have evaluated the adverse effects of atmospheric emissions on ecosystem health and function (e.g., Lyanguzova, 2017; Parviainen et al., 2024; Trubina et al., 2014). PTEs commonly analysed in emissions from such industries include Al, Cr, Cu, Fe, Pb, and Zn (Bozkurt, 2017; Brunialti & Frati, 2014; Formela, 2021; Nimis et al., 2000; Parviainen et al., 2019; Rangel-Osornio et al., 2022).

This study focused on assessing the effects of air pollution on lichen samples (*in situ* and transplants) in the surroundings of a waste recycling industry, particularly facilities processing tyres and Cu cables, in order to identify potential environmental impacts caused by atmospheric emissions of PTEs from the pollution source. We hypothesized that the recycling industry acts as the main source of atmospheric trace metal pollution in the study area, generating a spatial gradient of element accumulation in *Evernia prunastri* influenced by distance from the source and prevailing wind direction, and that *in situ* samples would accumulate higher concentrations than transplanted samples due to their longer exposure time. To test this hypothesis, the following specific objectives were established: (1) to determine the concentration of selected trace elements (Al, Cr, Cu, Fe, Pb and Zn) accumulated by *E. prunastri* in *in situ* and transplanted samples; (2) to calculate the enrichment factor in order to assess the influence of soil-derived inputs on the samples; (3) to evaluate atmospheric deposition patterns of each element at different distances and directions from the recycling industry; and (4) to identify areas with higher total contamination and the spatial distribution of toxic elements potentially affecting environmental quality.

## Material and methods

### Study area

**Fig. 1:**
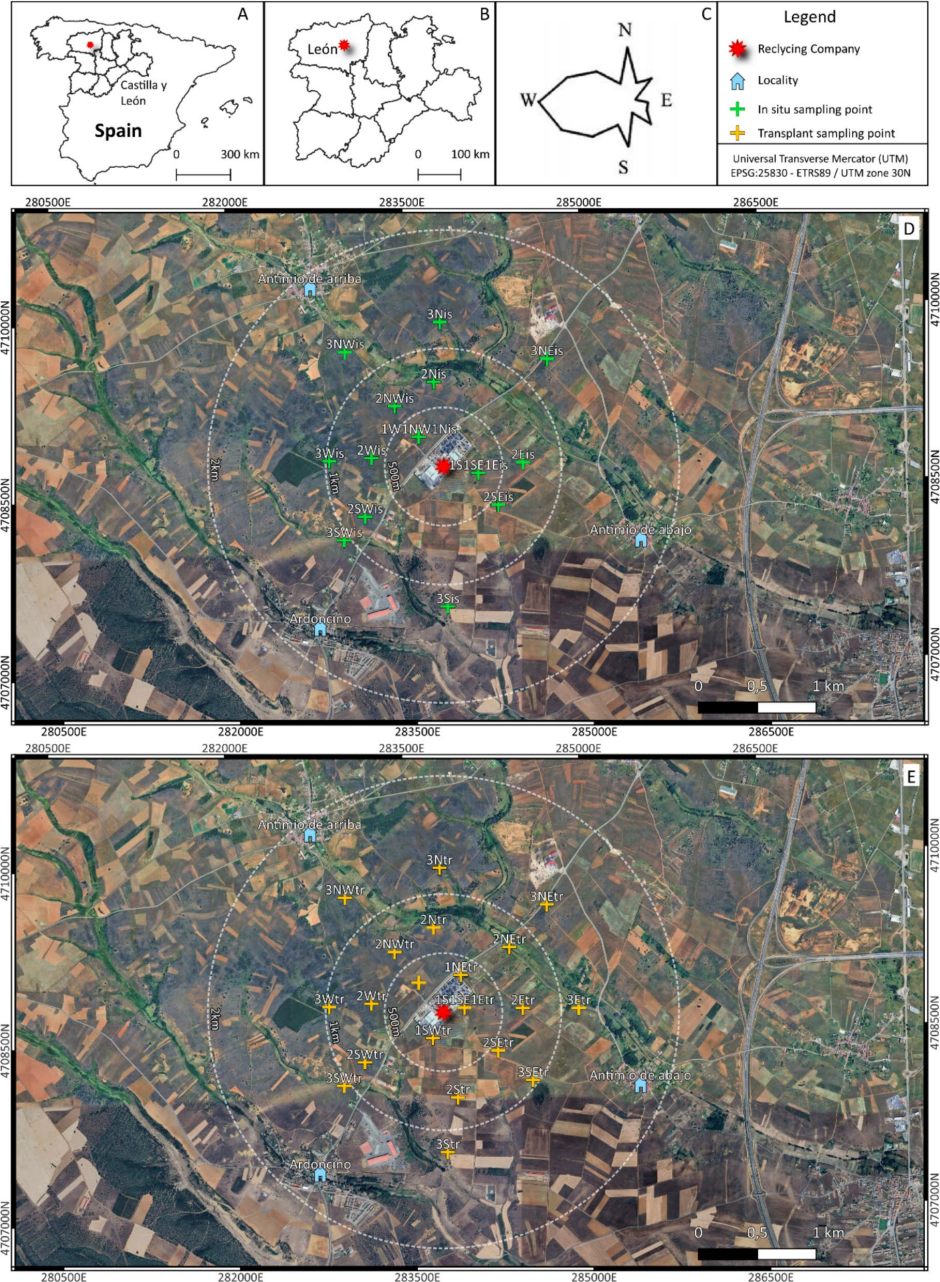
Map of the study area. (**A**) Geographic location of Castilla y León, (**B**) Geographic location of León in northwest Spain, (**C**) Direction from which the prevailing winds blow (Weather station—La Virgen de Camino), (**D**) Location of the *in situ* at sampling points, (**E**) Location of the transplant at sampling points

The study area is located at 12 km from León, in the northwest of the Iberian Peninsula, in Spain (42° 30′ 04″ N, 5° 37′ 53″ W) (Figs. 1A, B). The nearest localities are Ardoncino, Antimio de Arriba, and Antimio de Abajo. The study area covers approximately 3.14 km^2^, with a recycling industry situated at the centre. This recycling industry focuses mainly on recycling tyres and Cu cables, among other types of waste.

The area's orography consists of a plain with few elevations and an average altitude of 846 m. The prevailing winds blow from the W and S (Fig. 1C). The landscapes are characterised by active cereal crops, such as wheat and barley (towards Antimio de Abajo) and secondary vegetation (towards Antimio de Arriba) with species such as *Rosa* sp. (group of *R. canina* L.), *Thymus mastichina* (L.) L., and *Cytisus scoparius* (L.) Link.

The surveyed area presents a Mediterranean climate with pronounced droughts in July and August. The average annual temperature is 11 °C and the annual rainfall is 559 mm (Fick & Hijmans, 2017).

### Experimental design

To achieve representative, sampling points were arranged systematically, considering efficiency and statistical relevance. Data was collected at intervals along linear transects, suitable for identifying deposition gradients (Giordani et al., 2019).

Eight transects were established in cardinal directions (N, NE, E, SE, S, SW, W, NW). Along each transect, three sampling points were selected at distances of 0 m, 500 m and 1000 m approximately, from the recycling plant, which considered the central reference point. Due to insufficient separation, sampling points S-SE-E and N-NW-W (0 m) were unified, resulting in 20 stations (Figs. 1D and E).

### *Evernia prunastri* sampling

Native thalli of *E. prunastri* (*in situ*) were collected to assess the air quality of the survey area in recent years. Due to the influence of anthropogenic activities such as agriculture and industry, no sufficient material was found for analysis at the sampling points of NE, E, SE, SW and S at 0 m of the industry.

*In-situ* samples were manually collected from the branches of various phorophytes as *Rosa* sp. from the *R. canina* group, *T. mastichina*, and *C. scoparius*. The thalli collected had a uniform size (3 to 5 cm) and weight (200 g per point) and they were placed in sealed paper bags to avoid contamination or further deposition.

Transplants of *E. prunastri* have been widely used in studies on the accumulation of trace elements. These studies have demonstrated its high bioaccumulation capacity (Mallen-Cooper & Cornwell, 2020).

*E. prunastri* thalli were collected from a reference station situated near Candanedo de Boñar (42° 48′ 19″ N, 5° 20′ 47″ W). The reference site is characterized by *Quercus pyrenaica* Willd. forest under environmental conditions similar to those of the study area, although unaffected by atmospheric pollution. The lichens from the reference site exhibit healthy growth and well-developed thalli. In the absence of human activities, the *Q. pyrenaica* forest would naturally extend to the study area, as it represents the climatic climax stage in this region (Rivas-Martínez, 2011).

After collection, the samples were distributed into 65 mesh bags. The samples were transplanted around the recycling industry distributed in 20 sampling points with three samples in each one. Two of them were isolated immediately after collection to prevent alteration and serve as time zero (T0) controls. Additionally, three samples were returned to Candanedo de Boñar (reference station), forming a single reference point with three samples.

All transplants were positioned at a height of 1–1.5 m above ground level and exposed to atmospheric contaminants for a period of four months (August-November) to analyse de air quality in the survey area and compare with *in situ* samples.

### Laboratory analysis

At the end of the exposure period, transplant and *in situ* samples were transported to the laboratory. The lichen samples were dried at 40 ºC for 24 h, then they were cleaned of foreign objects such as tree bark and ground. The three samples collected at each point were pooled into a single composite sample to reduce laboratory analytical costs associated with processing each sample individually, meaning that only one sample per point is analysed, without replicates.

After cleaning, the samples were analysed in the Laboratory of Instrumental Techniques (LTI) at the University of León to determine the concentrations of six trace elements: Al, Cr, Cu, Fe, Pb, and Zn. The methodology proposed by Giordani et al. (2019) was followed, incorporating modifications made by the LTI at the University of León.

For elemental analysis, 0.5 g of each sample were digested with 10 ml of 65% HNO_3_, 3 ml of 37% HCl and 3 ml of 48% HF in an atmospheric pressure reflux digester. The digestion protocol included a temperature ramp from room temperature to 45 °C over 30 min, followed by a 1-min hold at 45 °C. Then, the temperature was increased to 65 °C over 25 min and held for 5 min. A further increase to 100 °C was applied over 15 min, followed by a 120 min hold at 90 °C. After cooling, the volume was brought up to 20 mL with Milli-Q water. Fe, Al, Cu, and Zn were analysed using ICP-OES (Atomic Emission Spectroscopy with Inductively Coupled Plasma) while Cr and Pb were measured using ICP-MS (Inductively Coupled Plasma Mass Spectrometry). The ICP-MS was calibrated with standards of 0, 2, 10, 50, 100, and 300 ppb and internal standards of 100 ppb scandium (Sc) and 10 ppb rhodium (Rh) and platinum (Pt) were added. Cr was measured using a helium gas collision cell at a flow rate of 4.2 ml/min, and lead at a flow rate of 1.5 ml/min. Samples analysed by ICP-OES were measured without dilution. The ICP-OES instrument was calibrated with a multi-element solution containing 0, 0.01, 0.05, 0.1, 0.5, 1 and 10 ppm of each element, with an additional 20 ppm standard included for Al and Fe. A 5 ppm solution of yttrium (Y) was used as an internal standard.

Analytical accuracy was assessed using the certified reference material IAEA-336 (*E. prunastri*) provided by the International Atomic Energy Agency. Recoveries for the analysed elements were within acceptable ranges: 102.50% for Al, 86.51% for Cr, 89.72% for Cu, 103.02% for Fe, 87.76% for Pb, and 97.37% for Zn, confirming the reliability of the analytical procedure. Relative standard deviations (%RSD) were below 5% for all analysed elements, indicating good analytical precision.

### Enrichment factor

The Enrichment Factor (EF) was calculated to evaluate the terrigenous vs. anthropogenic origin of trace elements in lichens. To provide a conservative estimate and reduce analytical costs, the EF was calculated by comparing the highest concentration measured for each element in lichen samples with the corresponding concentrations in soil samples collected in the study area at the end of the transplant exposure period. The soil samples were analysed using the same digestion and instrumental methods as the lichen samples (Klos et al., 2011; Mohamed et al., 2023). The formula is: EF = (X_L_/R_L_)/(X_S_/R_S_); where X_L_ is the concentration of the element of interest in the lichen, R_L_ is the concentration of the reference element in the lichen, X_S_ is the concentration of the element of interest in the soil, and R_S_ is the concentration of the reference element in the soil (Rudnick & Gao, 2003).

Al was used as the reference element for the soil (R_S_), as it is a major constituent of the Earth's crust and has limited metabolic significance in lichens (Bargagli, 1989). For the calculation of EF for Al, Fe was used as an additional reference element for the soil (Loppi et al., 1999).

EF values < 10 suggest soil enrichment., whereas EF values > 10 indicate an anthropogenic origin. Furthermore, EF values above 30 indicate a relevant anthropogenic contribution and confirming their tendency to behave as atmophile elements subject to long-range atmospheric transport (Loppi et al., 1999).

### Data analysis and mapping

To assess trace element accumulation in transplanted lichens, accumulation ratios were calculated by dividing the concentration of each element measured in exposed samples by the corresponding concentration at T0.

Before performing statistical analyses, the normality of the concentration data was assessed using the Shapiro–Wilk test. Among the analysed elements, only Al showed a normal distribution and was analysed without transformation. For the remaining elements, logarithmic transformations were applied to approximate normality. Given that some transformed datasets still showed marginal departures from normality, generalized linear mixed models (GLMMs) with a Gaussian family were used as a more flexible analytical framework, whereas linear mixed models (LMMs) were applied when normality assumptions were met. The significance of differences was assessed at *p* < 0.05.

To analyse the effects of distance from the recycling industry and exposure direction, LMMs or GLMMs with a nested design were applied, using element concentrations (numeric variable) as the response variable. Distance from the recycling industry (numeric variable), exposure direction (categorical variable), and their interaction were included as fixed effects. The nesting of monitoring stations into two groups according to the prevailing wind direction (W–E) was considered as a random effect. When non-significant terms were identified (*p* > 0.05), models were simplified by retaining only significant predictors (*p* < 0.05). Spearman’s rank correlation was additionally used to assess pairwise associations between elements using the original, non-transformed data, thereby avoiding potential distortions associated with data transformation.

Principal component analysis (PCA) was performed on the concentration data to explore relationships among elements and identify the main gradients in the dataset. In addition, hierarchical cluster analysis (HCA) was applied to group elements according to their similarity in accumulation patterns, using Euclidean distance as the dissimilarity measure and complete linkage as the clustering method. Studies have shown that the complete linkage method is a powerful grouping mechanism for classifying lichens, and that Euclidean distance is likewise an appropriate and effective measure of dissimilarity (Brunialti & Frati, 2007; Doǧrul Demiray et al., 2012). All analyses were performed in R version 4.5.1 (R Core Team, 2024).

In order to represent pollutant patterns spatially, the software SURFER v.8.0 was used to elaborate distribution’s maps. Kriging (geostatic autocorrelation of the nearest randomly placed value to produce an estimate of minimum least squares variance) was used as interpolation algorithm interpolation method (Bullo et al., 1995), since it has been found to be a very useful aid for examining hazardous situations from continuous point sources (Rangel-Osornio et al., 2022).This method generates a regular grid overlaid on the study area based on the concentrations of each element at the studied points (Giordani et al., 2019). Total contamination and toxic trace metals (Pb and Cr) spatial pattern were elaborated. For this, we used the values extracted from the *in situ* and transplanted samples of *E. prunastri* following the standardisation method of Gasparo et al. (1989).

## Results and discussion

### Trace elements bioaccumulation in *in situ* samples of *E. prunastri*

The content of trace elements in *E. prunastri in situ* samples, and soil samples collected around the recycling industry and the reference site and T0 samples is reported in Table 1.

**Table 1:** Concentrations of the six elements analysed in *E. prunastri* samples *in situ*, in the reference site, T0 and soil

	Al	Cr	Cu	Fe	Pb	Zn
1W1NW1N	616.74 ± 1.95	1.81 ± 0.06	132.83 ± 0.35	713.89 ± 3.99	22.34 ± 0.21	139.70 ± 0.52
2N	762.82 ± 2.44	1.51 ± 0.01	29.55 ± 0.04	577.35 ± 3.82	5.05 ± 0.02	39.11 ± 0.10
3N	508.12 ± 3.37	0.88 ± 0.02	12.23 ± 0.03	328.35 ± 3.83	2.19 ± 0.02	27.73 ± 0.04
3NE	773.17 ± 0.82	1.67 ± 0.03	12.60 ± 0.03	576.69 ± 5.48	4.07 ± 0.01	40.50 ± 0.11
2E	462.81 ± 0.26	1.03 ± 0.04	31.76 ± 0.08	432.50 ± 6.39	4.15 ± 0.01	56.94 ± 0.08
2SE	628.59 ± 0.34	2.67 ± 0.02	165.41 ± 0.21	697.22 ± 4.70	15.10 ± 0.09	95.04 ± 0.29
1S1SE1E	707.31 ± 3.04	3.30 ± 0.06	193.91 ± 0.19	975.71 ± 11.21	15.24 ± 0.05	162.30 ± 0.47
3S	523.61 ± 2.13	1.83 ± 0.01	48.28 ± 0.21	472.82 ± 4.91	3.87 ± 0.04	44.73 ± 0.30
2SW	550.33 ± 1.65	1.05 ± 0.02	38.19 ± 0.01	362.29 ± 0.29	4.36 ± 0.02	51.57 ± 0.10
3SW	677.05 ± 3.34	1.51 ± 0.01	27.61 ± 0.05	482.41 ± 7.31	3.26 ± 0.01	37.34 ± 0.18
2W	580.59 ± 1.54	1.25 ± 0.01	40.05 ± 0.12	383.36 ± 1.46	4.42 ± 0.03	43.25 ± 0.13
3W	470.06 ± 0.57	1.19 ± 0.01	56.52 ± 0.12	349.18 ± 2.56	4.57 ± 0.01	35.82 ± 0.14
2NW	612.56 ± 1.82	1.24 ± 0.02	24.85 ± 0.03	408.19 ± 2.33	3.28 ± 0.03	39.63 ± 0.18
3NW	495.95 ± 1.88	1.37 ± 0.01	13.07 ± 0.04	327.51 ± 2.58	1.62 ± 0.01	23.76 ± 0.17
Reference site	403.31 ± 0.24	1.18 ± 0.01	4.28 ± 0.00	211.20 ± 1.10	0.79 ± 0.01	9.33 ± 0.05
T0	503.73 ± 0.04	1.03 ± 0.01	2.47 ± 0.01	274.22 ± 3.73	0.75 ± 0.01	8.77 ± 0.03
Soil	11574.40 ± 103.63	101.47 ± 1.20	32.60 ± 0.07	23420.70 ± 427.74	27.88 ± 0.10	55.40 ± 0.47

The concentrations are shown in mg/kg ± SD of technical analytical replicates. Samples with number 1 (0 m); 2 (500 m); 3 (1000 m)

The high EF values (Table 2) indicate the impact of the anthropogenic source on the lichen chemistry for Cu (EF = 97.34), Zn (EF = 47.96) and Pb (EF = 15.04). Cu and Zn present values above 30 confirming their tendency to behave as atmophile elements subject to long-range atmospheric transport. Nevertheless, it can be considered Fe (EF = 0.68), Cr (EF = 0.53) and Al (EF = 2.71) as terrigenous.

**Table 2:** Enrichment factor (EF) values for *in situ* and transplanted (tr) lichen samples collected within the study area

	Al	Cr	Cu	Fe	Pb	Zn
EF *in situ*	2.71	0.53	97.34	0.68	15.04	47.96
EF tr	0.73	0.62	124.99	0.64	12.46	37.04

The elemental concentrations in T0 and reference samples were more like non-contaminated environments (Bargagli & Nimis, 2002). In the rest of the sites, the elemental concentrations, except for some points for Cr, were higher than those found in the reference site samples indicating a clear accumulation of elements.

The maximum concentration of Al obtained for *in situ* lichen samples was 773.17 mg/kg, while the minimum was 470.06 mg/kg. Based on the maximum value, this area corresponds to a medium naturalness level in terms of environmental alteration (Bargagli & Nimis, 2002). According to Bozkurt (2017), high Al concentrations are typical in rural areas. The Al accumulation in this study is lower when compared to the study of Pacheco et al. (2008) (1337 mg/kg), where *E. prunastri in situ* samples were also measured at an industrial complex near Lisbon. Conti et al. (2009) used *Usnea barbata* (L.) Weber ex F.H. Wigg. in low contamination areas in southern Patagonia and reported higher Al values, with a maximum of 2107 mg/kg. Similarly, Brunialti and Frati (2007) measured high levels of Al in the lichen *Xanthoria parietina* (L.) Th.Fr. in an industrial area on the Adriatic coast of Italy.

For Cr, the maximum concentration was 3.30 mg/kg, while the minimum was 0.88 mg/kg. According to the scale by Bargagli and Nimis (2002), this corresponds to medium naturalness. These values are even lower than those recorded for rural areas by Bozkurt (2017). The Cr concentrations are lower than those registered by Pacheco et al. (2008) at 6 months of exposure (3.54 mg/kg) and by Brunialti and Frati (2007) (2.7 mg/kg). The Cr data were more similar to those found in *Usnea barbata* (Conti et al., 2009). Furthermore, these values were similar to those found by Fuga et al. (2008) in non-contaminated areas to the east of Brazil for native *Canoparmelia texana* (Tuck.) Elix & Hale. Mota et al. (2025) reported comparable concentrations (0.92—2.25 mg/kg) in expanding residential and industrial areas, whereas substantially higher levels (10.17—31.14 mg/kg) were observed in zones characterized by intense industrial activity (chemical and petrochemical industries) and heavy traffic, based on native *C.texana* in São Paulo (Brazil).

Cu showed a maximum concentration of 193.91 mg/kg and a minimum of 12.60 mg/kg. These values correspond to an area of very high alteration, and they are considerably higher than the maximum value recorded for Italy of 161 mg/kg (Bargagli & Nimis, 2002). These Cu concentrations were much higher than those obtained by Pacheco et al. (2008) at 6 months of exposure (12.60 mg/kg) and Brunialti and Frati (2007) (9.04 mg/kg). These concentrations are also considerably higher than those reported for *Usnea barbata* (2.46 mg/kg) (Conti et al., 2009). A similar Cu concentration was registered for urban areas by Bozkurt (2017). The high Cu values found in this study are typical of mining areas, according to the Cu concentration accumulated in *X. parietina* observed by Parviainen et al. (2024) (948.00 mg/kg) in the active mining area located in Huelva, in SW Spain.

The maximum and minimum concentrations of Fe were 975.71 mg/kg and 327.51 mg/kg, respectively. According to the scale by Bargagli and Nimis (2002), these values correspond to a zone of medium naturalness. The highest values, found at 0 m from the industrial area, resemble those found by Pacheco et al. (2008) at 6 months of exposure (1265 mg/kg). In other studies (Bozkurt, 2017; Conti et al., 2009; Fuga et al., 2008; Nimis et al., 2000), the Fe values were like those reported in this study and correspond to rural areas. However, these concentrations were lower than those reported by Parviainen et al. (2024) in active mining areas (8828 mg/kg).

For Pb, the maximum concentration obtained was 22.34 mg/kg, while the minimum was 1.62 mg/kg. These values correspond to an area with high to medium naturalness alteration (Bargagli & Nimis, 2002). Our results were higher than those obtained by Loppi et al. (2000) for *Flavoparmelia capetara* (L.) Hale *in situ* samples (1.92 mg/kg) in the closest point (50 m) to a solid waste incinerator in central Italy. According to the monitoring by Brunialti and Frati (2007), the Pb concentrations are like those obtained in 1996 (7.66 mg/kg) and higher than those recorded in 2003 (2.43 mg/kg) in oil combustion electric power plants. However, these concentrations were lower than those reported by Parviainen et al. (2024) (63.00 mg/kg) in lichens from an active mining area; but in general, our data correspond to concentrations reported rural environments (Bozkurt, 2017).

Zn was accumulated from 162.30 mg/kg (maximum) to 23.76 mg/kg (minimum). Considering the concentrations of Zn, which are higher at 0 m from the industrial area, the area is classified as highly to very highly altered. However, the concentrations at 500 m and 1 km place the area at medium naturalness (Bargagli & Nimis, 2002). Mota et al. (2025) reported Zn concentrations (169.63 mg/kg) comparable to the maximum value observed in our study in areas characterized by intense chemical and petrochemical industrial activity and heavy traffic. When comparing these data with those obtained by Pacheco et al. (2008) in contaminated areas for *E. prunastri*, the Zn values reported in this study were very similar (36.15—61.77 mg/kg). The same occurs in the study by Conti et al. (2009) for *Usnea barbata*, where the data for Zn in low contamination areas are lower. The data are like those obtained in studies near industrial areas (Frati et al., 2005; Nimis et al., 2000).

### Trace elements bioaccumulation in *E. prunastri* transplants

The content of trace elements in *E. prunastri* samples transplanted around the recycling industry is reported in Table 3.

**Table 3:** Concentration of the six elements analysed at each sampling point for *E. prunastri* transplants refer to the industry

	Al	Cr	Cu	Fe	Pb	Zn
1W1NW1N	607.14 ± 2.64	1.62 ± 0.03	153.40 ± 0.88	505.62 ± 5.67	10.73 ± 0.07	53.10 ± 0.14
2N	500.62 ± 2.15	1.43 ± 0.01	17.31 ± 0.10	298.82 ± 2.89	1.77 ± 0.01	14.64 ± 0.05
3N	613.14 ± 1.28	1.24 ± 0.03	11.33 ± 0.05	366.60 ± 3.70	1.71 ± 0.01	14.43 ± 0.05
2W	590.74 ± 1.03	1.49 ± 0.02	38.98 ± 0.14	357.53 ± 4.83	3.72 ± 0.02	20.05 ± 0.06
3W	451.78 ± 1.25	1.29 ± 0.01	26.01 ± 0.11	292.05 ± 2.93	2.64 ± 0.01	16.52 ± 0.05
2NW	423.93 ± 0.56	1.33 ± 0.02	27.68 ± 0.09	278.05 ± 1.96	2.62 ± 0.01	18.53 ± 0.04
3NW	531.58 ± 0.54	2.88 ± 0.03	7.87 ± 0.02	317.64 ± 3.11	1.21 ± 0.01	12.13 ± 0.06
1NE	475 ± 1.08	2.48 ± 0.11	67.05 ± 0.21	455.18 ± 5.02	5.83 ± 0.01	46.95 ± 0.24
2NE	543.43 ± 0.87	1.19 ± 0.02	24.15 ± 0.07	344.29 ± 0.79	2.64 ± 0.03	17.72 ± 0.07
3NE	603.09 ± 0.54	1.51 ± 0.07	14.72 ± 0.04	431.54 ± 7.98	2.25 ± 0.02	20.34 ± 0.12
1S1SE1E	713.68 ± 1.67	2.34 ± 2.34	124.19 ± 0.48	922.03 ± 5.73	7.96 ± 0.07	126.53 ± 0.26
2E	575.05 ± 1.28	1.39 ± 0.05	27.21 ± 0.06	389.68 ± 3.69	2.35 ± 0.02	21.82 ± 0.08
3E	622.75 ± 0.61	1.43 ± 0.04	13.86 ± 0.02	378.39 ± 6.31	1.66 ± 0.02	16.39 ± 0.08
2SE	598.71 ± 1.32	1.66 ± 0.04	210.77 ± 0.54	417.50 ± 4.95	9.04 ± 0.05	49.52 ± 0.18
3SE	655.21 ± 2.45	1.44 ± 0.04	63.47 ± 0.27	425.81 ± 6.4	3.47 ± 0.02	25.81 ± 0.24
2S	638.21 ± 0.98	1.40 ± 0.02	67.44 ± 0.21	433.95 ± 3.86	4.86 ± 0.04	26.68 ± 0.09
3S	477.71 ± 1.01	1.62 ± 0.04	17.7 ± 0.03	274.28 ± 3.70	1.89 ± 0.03	13.74 ± 0.06
1SW	749.98 ± 0.39	1.68 ± 0.05	208.84 ± 0.58	505.56 ± 1.67	22.52 ± 0.08	80.99 ± 0.58
2SW	451.96 ± 0.50	0.86 ± 0.01	17.75 ± 0.02	255.22 ± 2.67	1.99 ± 0.01	14.23 ± 0.05
3SW	525.67 ± 0.54	1.32 ± 0.03	16.62 ± 0.01	309.44 ± 3.73	2.11 ± 0.01	15.97 ± 0.11

The concentrations are shown in mg/kg ± SD of technical analytical replicates. Samples with number 1 (0 m); 2 (500 m); 3 (1000 m)

The EF values (Table 2) for transplanted lichens indicate anthropogenic enrichment for Pb (EF = 12.46), Zn (EF = 37.04), and especially for Cu (EF = 124.99), whereas this is not observed for Al, Fe and Cr.

Most *E. prunastri* transplants exposed in the study area showed accumulation ratios higher than 1 relative to T0 samples (Table 4), indicating the accumulation of PTEs in their thalli during the exposure period, except for Al, Cr, and Fe in some sampling points. The low values of accumulation may be due to the cleaning action of rainwater. It is known that rain is a rich source of elements for lichens, but it can also eliminate or "wash" the particles deposited on the surface of the thallus (Kularatne & De Freitas, 2013). It was also observed that the reference site samples did not accumulate Al or Fe, while a slight increase in Cu concentrations was detected after transplantation. This may suggest the influence of seasonal variations affecting both elemental concentrations and lichen physiology throughout the year (Prussia & Killingbeck, 1991). However, Cu concentrations at the reference site were substantially lower than those measured in samples collected near the recycling industry, indicating a marked accumulation of this element within the study area.

**Table 4:** Accumulation ratios of trace elements in exposed *Evernia prunastri* transplants and reference site samples relative to their corresponding concentrations at time 0 (T0)

	Al	Cr	Cu	Fe	Pb	Zn
1W1NW1N	1.21	1.57	62.11	1.84	14.30	6.05
2N	**0.99**	1.38	7.01	1.09	2.36	1.67
3N	1.22	1.20	4.59	1.34	2.28	1.65
2W	1.17	1.44	15.78	1.30	4.96	2.29
3W	**0.90**	1.25	10.53	1.07	3.52	1.88
2NW	**0.84**	1.28	11.21	1.01	3.49	2.11
3NW	1.06	2.78	3.19	1.16	1.61	1.38
1NE	**0.94**	2.39	27.15	1.66	7.77	5.35
2NE	1.08	1.15	9.78	1.26	3.51	2.02
3NE	1.20	1.46	5.96	1.57	3.00	2.32
1S1SE1E	1.42	2.26	50.28	3.36	10.60	14.43
2E	1.14	1.34	11.02	1.42	3.14	2.49
3E	1.24	1.38	5.61	1.38	2.21	1.87
2SE	1.19	1.60	85.33	1.52	12.04	5.65
3SE	1.30	1.39	25.70	1.55	4.62	2.94
2S	1.27	1.35	27.30	1.58	6.47	3.04
3S	**0.95**	1.56	7.17	1.00	2.52	1.57
1SW	1.49	1.63	84.55	1.84	30.00	9.23
2SW	**0.90**	**0.83**	7.19	**0.93**	2.65	1.62
3SW	1.04	1.27	6.73	1.13	2.81	1.82
Reference site	**0.80**	1.14	1.73	**0.77**	1.05	1.06

Samples with number 1 (0 m); 2 (500 m); 3 (1000 m)

The maximum and minimum concentrations obtained for Al were 749.98 mg/kg and 423.93 mg/kg, respectively. According to the scale by Bargagli and Nimis (2002), this area is classified as having high/medium naturalness. When compared with another study where *E. prunastri* was transplanted for 3 months in urban areas, the values are similar (628 mg/kg) (Frati et al., 2005). However, they are lower than those obtained for *Pseudevernia furfuracea* (L.) Zopf after 3 months of exposure at 500 m of a biomass power plant (1431.00 mg/kg) (Lucadamo et al., 2016) and for *Usnea ceratina* Ach. after 4 months of exposure around a paper industry (499.40—1188.40 mg/kg) (Rangel-Osornio et al., 2022).

The maximum concentration of Cr was 2.88 mg/kg, and the minimum was 0.86 mg/kg. In general, the data corresponds to high naturalness, however some samples showed high values of Cr and therefore correspond to areas of medium naturalness (Bargagli & Nimis, 2002). Compared with previous transplant studies using *E. prunastri* in industrial, urban, and rural environments, the concentrations observed in our study fall within the range typically reported for urban areas (1.2—2.3 mg/kg) (Conti et al., 2004; Frati et al., 2005). Similarly, Boonpeng et al. (2023) reported comparable Cr concentrations (0.8—2.7 mg/kg) accumulated in thalli of *Parmotrema tinctorum* (Despr. ex Nyl.) Hale after two months of exposure in an expanding industrial area in Laem Chabang municipality (Thailand). Based on these concentration ranges, similar environments have been classified as transitional industrial zones (Cercasov et al., 2002; Paoli et al., 2011).

Regarding Cu, the maximum concentration reached 210.80 mg/kg, while the minimum was 7.9 mg/kg. These values corresponded to a very high alteration zone, exceeding the maximum recorded for Italy (161 mg/kg) (Bargagli & Nimis, 2002). The Cu concentrations are only reached at the points closest to the industry, decreasing as the distance increases, classifying the area as one of medium alteration (Bargagli & Nimis, 2002). These concentrations were significantly higher than those reported for *E. prunastri* transplants in industrial areas by Conti et al. (2004) (2.5–6.3 mg/kg), Frati et al. (2005) (12.1 mg/kg) and Paoli et al. (2015) (5.4 mg/kg). Similarly, these concentrations were higher than those reported for *E. prunastri* transplants near a municipal solid waste landfill (15.10 mg/kg) in central Italy (Nannoni et al., 2015) and reported for *P. tinctorum* transplant in an expanding industrial area in Laem Chabang municipality (Thailand) (1.9—6.3 mg/kg) (Boonpeng et al., 2023).

For Fe, the maximum and minimum concentrations were 922 mg/kg and 255 mg/kg, respectively. If we consider the maximum concentration, we are dealing with a zone of low naturalness/alteration, although most of the data corresponds to high naturalness areas (Bargagli & Nimis, 2002). These values are normal for industrial urban areas (Frati et al., 2005) and slightly lower for areas with metallurgical industries (Cercasov et al., 2002), both for *E. prunastri* transplant. However, our Fe accumulation data were slightly higher than those reported by Boonpeng et al. (2023) (179.00—498.00 mg/kg) for transplants of *P.tinctorum* in an expanding industrial area in Thailand. Our data are also comparable to those reported for *U. ceratina* (155.50—739.10 mg/kg) and for *Flavopunctelia praesignis* (Nyl.) Hale (267.60—808.00 mg/kg) both transplanted around a paper industry in Morelia (Mexico) (Rangel-Osornio et al., 2022).

Pb concentrations ranged from 1.21 to 22.52 mg/kg, spanning values classified between medium and very high naturalness according to Bargagli and Nimis (2002). Except for the maximum concentration, Pb levels were comparable to those reported for *E. prunastri* transplants in urban and industrial areas (9.40 mg/kg) (Frati et al., 2005) and in around of a municipal solid waste landfill (15.40 mg/kg) (Nannoni et al., 2015). Compared with other transplant studies using *E. prunastri* across industrial, urban, and rural environments, the concentrations observed in our study were slightly higher than those reported for industrial areas (3.50—7.20 mg/kg) (Conti et al., 2004). Nevertheless, Pb accumulation remained substantially lower than that recorded after four months of exposure around metallurgical industries (45.00 mg/kg) (Cercasov et al., 2002).

Zn concentrations ranged from 12.13 to 126.53 mg/kg. Only the maximum concentration corresponded to an area classified as highly environmentally altered, whereas the remaining values generally fell within zones of high naturalness (Bargagli & Nimis, 2002). These concentrations are comparable to those reported for *E. prunastri* transplants near a municipal solid waste landfill (78.70 mg/kg) (Nannoni et al., 2015) and in metallurgical industrial areas (74.40 mg/kg) (Cercasov et al., 2002). However, they were slightly higher than concentrations reported for urban areas in central Italy (36.70 mg/kg) (Frati et al., 2005) and for expanding industrial areas in Thailand (21.00—37.00 mg kg⁻^1^) (Boonpeng et al., 2023), based on *E. prunastri* and *P. tinctorum* transplants, respectively. Zn concentrations observed in our study were also similar to the range reported for *Usnea ceratina* (18.70—94.20 mg/kg) transplanted around a paper industry (Rangel-Osornio et al., 2022).

### Spatial distribution of the elements

**Fig. 2:**
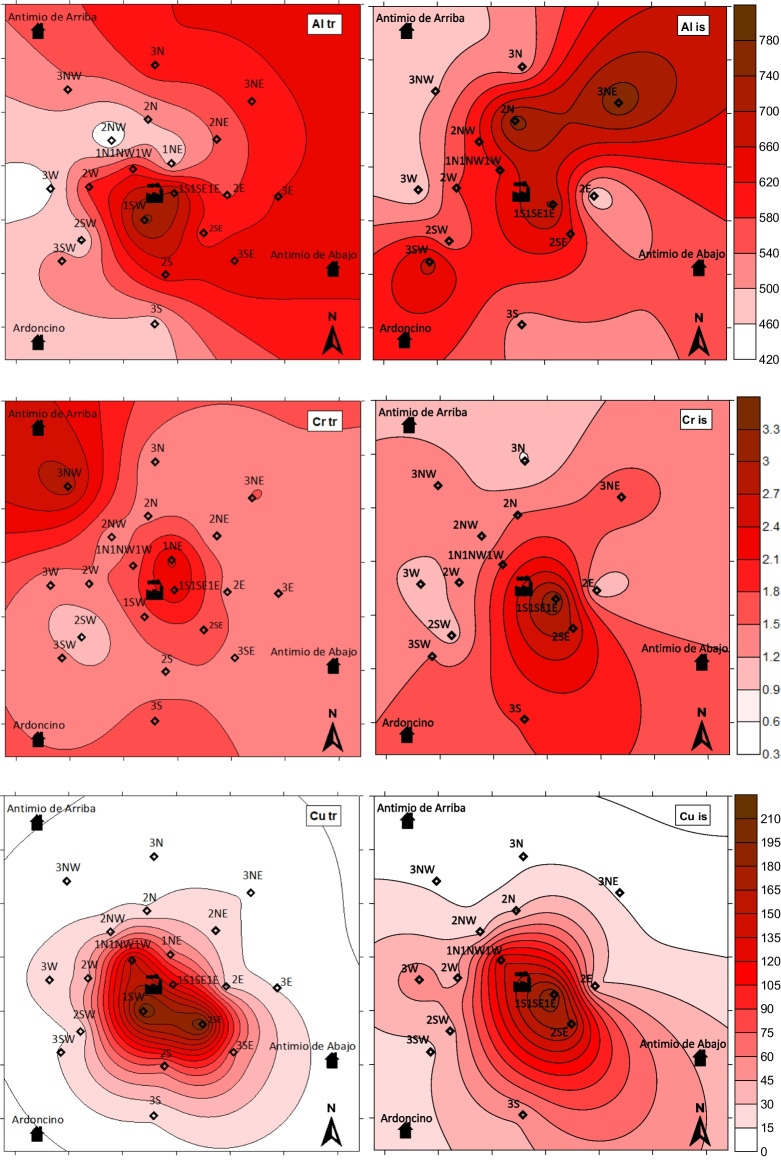

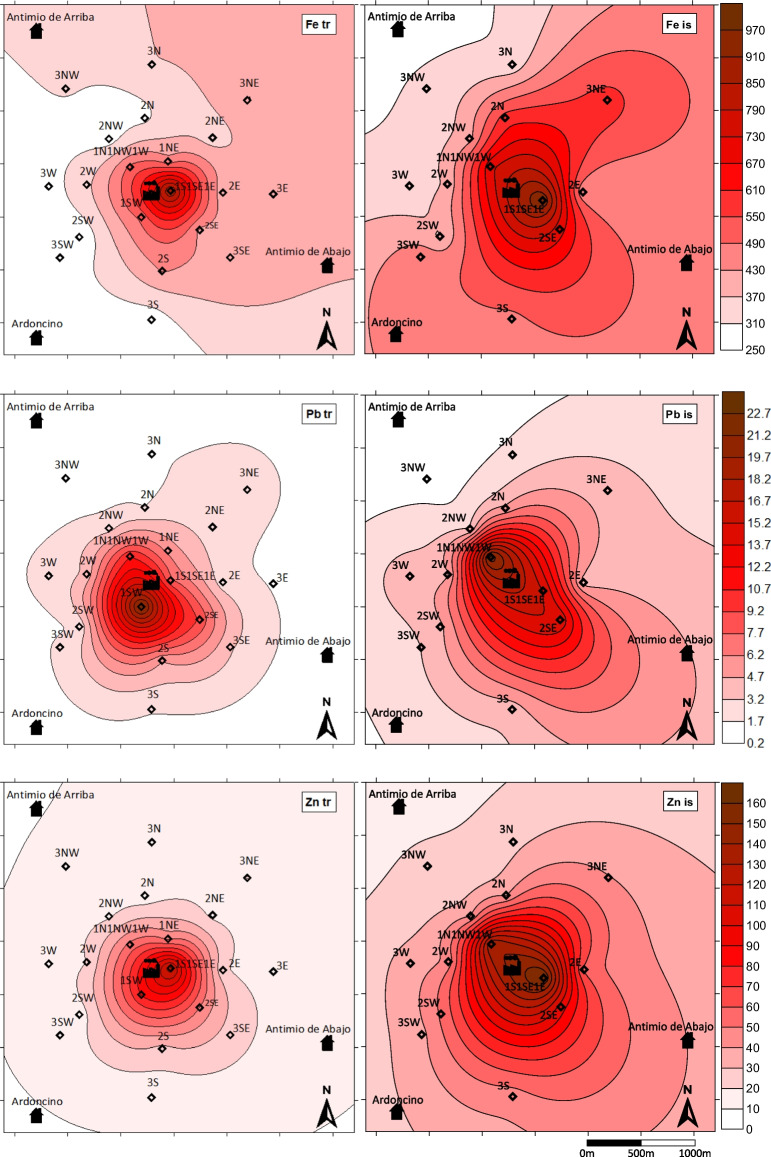
Spatial distribution maps of the six trace elements accumulated by *Evernia prunastri* transplants (maps on the left) and *in situ* (maps on the right) around the recycling industry. Darker colours indicate higher contents of trace elements (mg/kg)

**Fig. 3:**
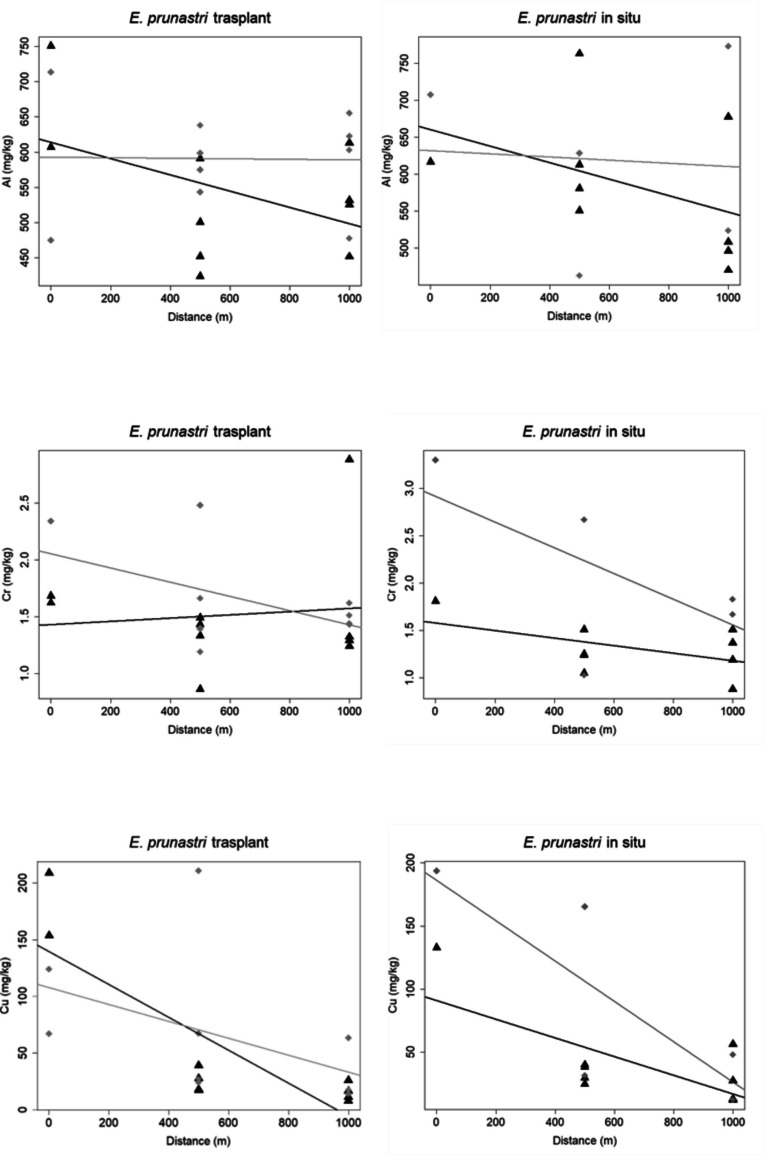

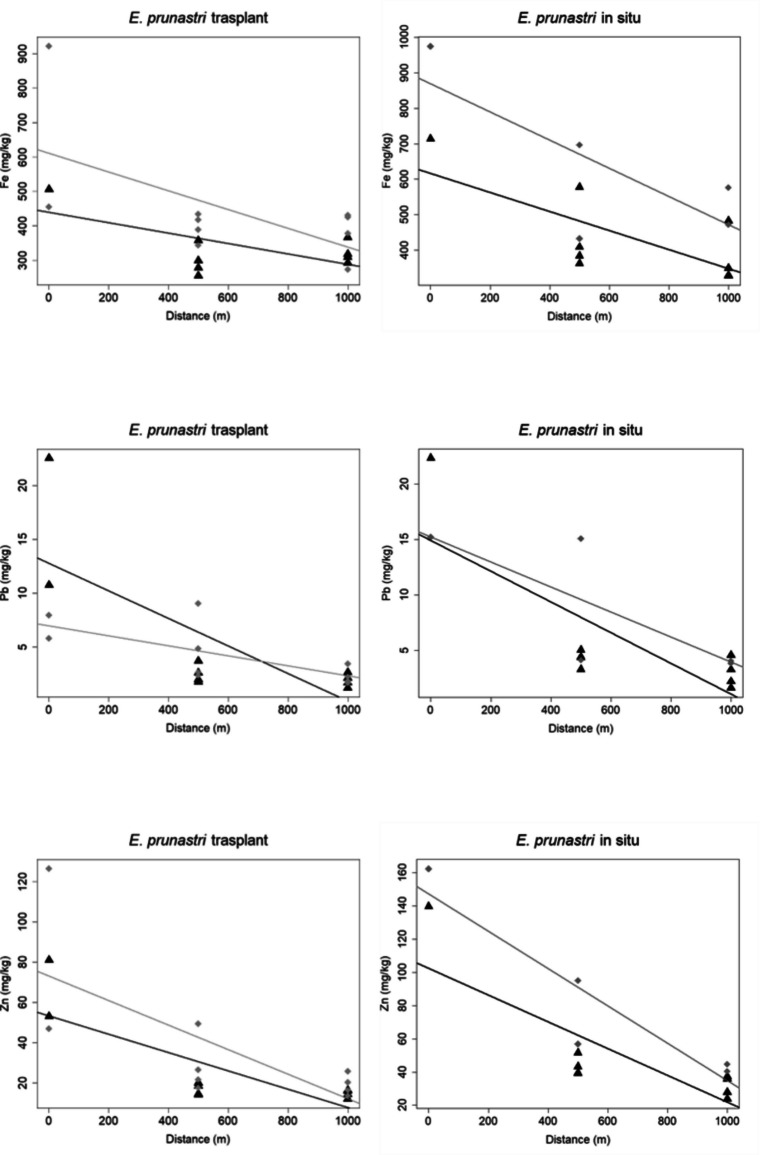
Relationship between the concentration of the six trace elements accumulated by *E. prunastri* transplants (on the left) and *in situ* (on the right) samples with the distance at the recycling industry. ▲: sampling points against prevailing winds. ◆: sampling points situated upwind of the prevailing winds

The maps in Fig. 2 show the distribution of the concentrations of the six analysed elements. The relationship between the concentration and the distance to the recycling industry, considering the action of prevailing winds, is shown in Fig. 3.

The distance from the pollution source influenced the concentrations of the elements, as well as their distribution. Generally, higher concentrations in transplanted samples were observed near to the recycling industry at 0 to 500 m, except for Cr. A similar distribution occurs in the *in situ* samples. This pattern of pollutant dispersion is like that observed by Rangel-Osornio et al. (2022) in a study around a paper industry and by Loppi et al. (2000) around a solid waste incinerator, where the concentration of trace elements decreases with the distance from the industry. In many cases, significantly elevated concentrations are found near the sources of contamination compared to more distant areas (Nash, 2008).

Regarding Al, the highest value in the lichen transplants is at station at 0 m while, in the *in situ* samples, it is found at NE and N at 1000 m from the recycling industry. In both lichen transplants and *in situ* samples, a dispersion pattern towards the NE can be observed. However, the relationship of this element to distance from the industry was not significant in lichen transplants (*p* = 0.27; R^2^ = 0.067) neither *in situ* samples (*p* = 0.34; R^2^ = 0.075). Furthermore, the effect of orientation was also not significant for both sampling techniques (lichen transplants; *p* = 0.25), *in situ* samples; *p* = 0.58). The accumulation pattern of the *in situ* samples could be related to the fact that these lichens have been exposed to this element for years. The presence of crops in the surrounding or Al natural abundance in the Earth's crust may influence the samples (Loppi & Paoli, 2015). Furthermore, as previously discussed, the low enrichment factor observed for Al (EF = 2.71) suggests that this element is predominantly derived from soil inputs, with only a minor contribution from industrial emissions.

The highest concentrations of Cr in lichen transplants were registered around the industry but significant differences in relation to the distance were not observed (*p* = 0.53). In *in situ* samples, the highest concentration is at the NW at 1000 m from the recycling industry, with a significant difference in relation to the orientation (*p* = 0.03) but not regarding distance (*p* = 0.12). This specific anomaly could be due to soil enrichment (EF: 0.53) or contamination from another source. Cr is emitted by metallurgical, chemical, paper industries (Nordberg et al., 2015) and solid waste incinerator (Loppi et al. 2000). Other sources include traffic emissions and coal combustion (Bozkurt, 2017). Cr can enter the human body through food chains, accumulating over time and causing cardiovascular, nervous, gastrointestinal, and respiratory problems (Singh & Prasad, 2015).

Regarding Cu, both in lichen transplants and *in situ* samples, the highest concentrations are found close to the industry and decrease with distance. This is also confirmed by the strong significant relationship between concentration and distance from the recycling industry in both lichen transplant (*p* < 0.001) and *in situ* samples (*p* = 0.00463). In particular, Cu concentration seems more pronounced in the *in situ* samples located in the SE orientation, due to the action of prevailing winds, however significant differences were not observed (*p* = 0.26). Furthermore, neither the transplants nor the *in situ* samples show soil enrichment (*in situ* EF: 97.34; lichen transplanted EF: 124.99). Consequently, the industry releases significant amounts of copper into the atmosphere, leading to its deposition on lichens. Cu is released into the environment by metal and electrical industries, combustion processes, wood preservation, boilers, fertilisers, and pesticides. The source of this element in the study area may be associated with recycled cables, which contain conductive metals such as Cu and can release this element into the environment during the shredding process, as well as with tire wire particles, which also contain high copper concentrations (Kang et al., 2025; Yang et al., 2011).

Fe showed the highest concentrations near the studied recycling industry, decreasing with distance in both lichen transplants and *in situ* samples. The relationship between Fe concentrations and distance from the recycling industry was statistically significant for both lichens transplanted and *in situ* samples (*p* = 0.00912; *p* < 0.001, respectively). In addition, a dispersion pattern predominantly towards the E was observed, likely influenced by the prevailing westerly winds, with a significant effect of orientation in *in situ* samples (*p* = 0.0081476). However, Fe and Al exhibited a similar spatial distribution and were strongly associated, suggesting a predominantly terrigenous origin, as these elements are major constituents of soil-derived particles (Loppi & Paoli, 2015; Loppi et al. 2000). This interpretation is further supported by the EF values below than 1 in both *in* *situ* and transplanted lichens, indicating the absence of anthropogenic enrichment and confirming a soil origin. Therefore, the observed spatial pattern may therefore reflect the emission, transport, and resuspension of soil and dust particles in the study area. In this context, recycling activities, including vehicle movement, handling of materials, and mechanical processing, may contribute indirectly to increased atmospheric Fe concentrations through the resuspension of contaminated dust rather than representing a primary emission source (Formela, 2021). Although anthropogenic activities such as metallurgical, combustion, and construction processes can emit iron (Bozkurt, 2017), the strong covariance with aluminium and the low EF values suggests that soil-derived particles represent the dominant source in the present study.

The concentration of Pb decreases with distance from the industry, in both transplants and *in situ* samples, indicating its origin as a pollutant from the contamination source. This is also confirmed by the significant relationship between concentration and distance from the recycling industry in both lichen transplant (*p* < 0.001) and *in situ* samples (*p* < 0.001). Regarding orientation, it can be observed a pattern toward SE, however significant differences were not found in any of them, lichen transplant (*p* = 0.47) nor *in situ* samples (*p* = 0.46). Similar pattern distribution was found by Loppi et al. (2000) in a round of a solid waste incinerator. The primary source of Pb release is associated with vehicle traffic or industrial engines (Brunialti & Frati, 2007; Nimis et al., 2000). However, this was more significant in the past, when lead-based fuels were used (Bozkurt, 2017).

According to our results, Zn emissions appear to originate from the recycling industry, with concentrations decreasing as distance from it increases. The relationship between Zn concentration and distance from the recycling industry was statistically significant for both lichen transplants (*p* < 0.001) and *in situ* samples (*p* < 0.001). As observed for the other elements, *in situ* samples exhibited higher Zn concentrations than lichen transplants. In addition, a spatial dispersion pattern predominantly towards the east was observed, likely influenced by the prevailing westerly winds, with a significant association between Zn concentration and wind direction (*p* = 0.0387). The main sources of Zn emissions are metal industries, vehicle emissions, wood preservation, fertilisers, pesticides, and tyre particles (Adriano, 2001). Zinc oxide is used in tyre manufacturing as an activator of vulcanisation (Li et al., 2010). Vulcanisation is a rubber enrichment process that significantly improves its properties, such as abrasion resistance and thermal stability. However, vulcanised rubber is not biodegradable and cannot be easily processed, posing significant challenges in managing and recycling this type of waste. Today, the amount of devulcanised rubber is minimal, leading to the release of enrichment compounds into the environment during recycling (Formela, 2021).

### Trace elements pattern

Tables 5 and 6 present the Spearman's rank correlation values for the transplants and *in situ* samples, respectively. Correlations are observed between the following groups of elements: Al–Fe, Cu-Pb, Cu–Zn, Fe-Zn, Pb–Zn. These correlations were similar to those reported in other studies for *Xanthoria parietina* (Bozkurt, 2017).

**Table 5:** Spearman’s correlations coefficient among the different elements analysed in *E. prunastri* transplants

	Al	Cr	Cu	Fe	Pb	Zn
Al	1					
Cr	0.315	1				
Cu	0.313	0.351	1			
Fe	0.776^**^	0.556^*^	0.580^*^	1		
Pb	0.359	0.354	0.954^**^	0.667^*^	1	
Zn	0.552^*^	0.430	0.871^**^	0.857^**^	0.911^**^	1

*Correlation is significant at *p* < 0.05; **Correlation is significant at *p* < 0.01 (two-tailed)

**Table 6:** Spearman’s correlations coefficient among the different elements analysed in *E. prunastri in situ*

	Al	Cr	Cu	Fe	Pb	Zn
Al	1					
Cr	0.653^*^	1				
Cu	0.063	0.532^*^	1			
Fe	0.749^**^	0.794^**^	0.525^*^	1		
Pb	0.353	0.459	0.819^**^	0.674^*^	1	
Zn	0.270	0.497	0.727^**^	0.709^**^	0.714^**^	1

*Correlation is significant at *p* < 0.05; **Correlation is significant at *p* < 0.01 (two-tailed)

**Fig. 4:**
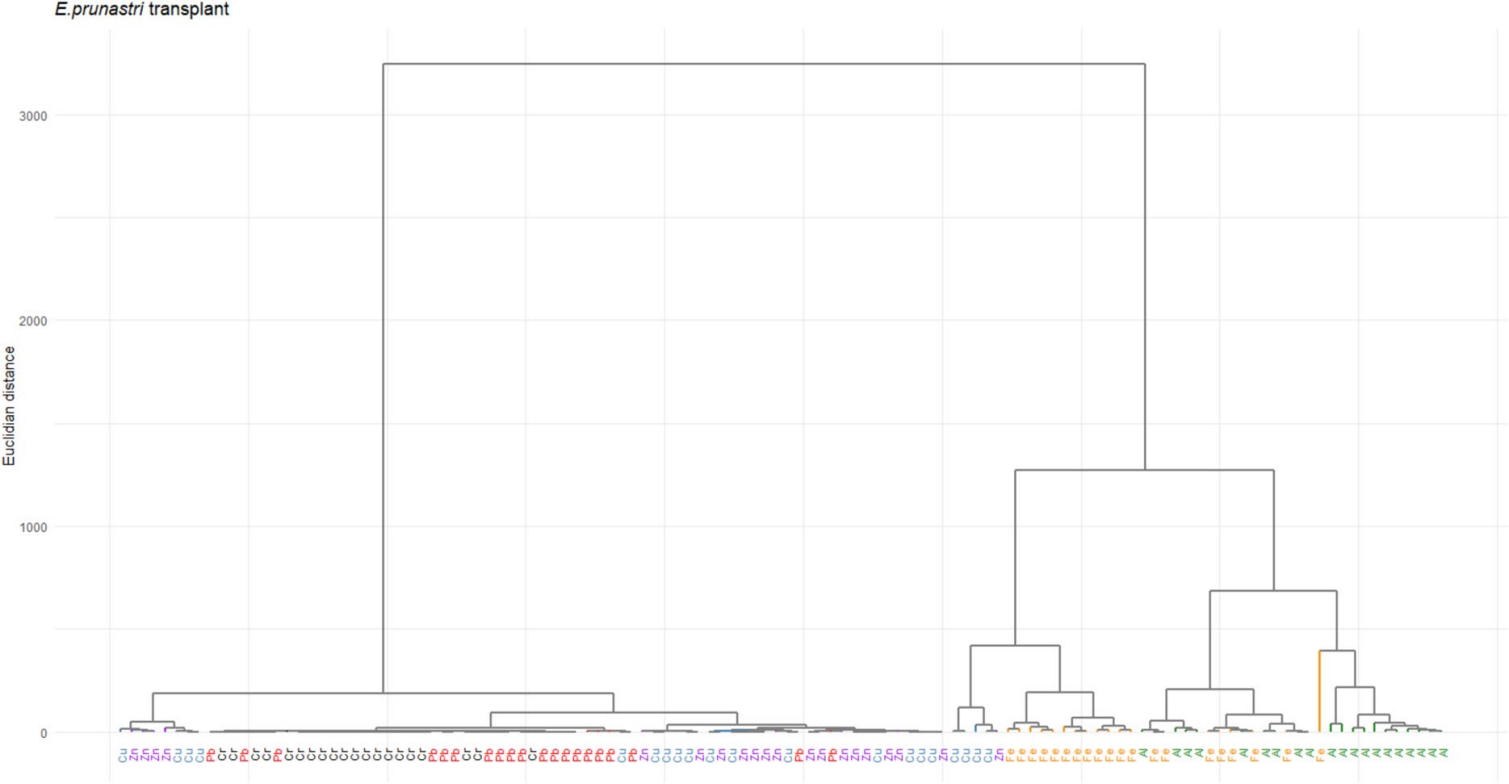

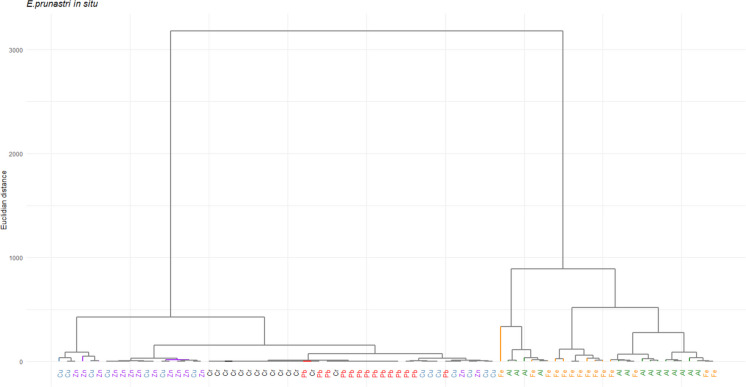
Cluster dendrograms of the trace elements in transplant samples (up) and *in situ* samples (down) from the study area

Regarding the classification of elements shown in the dendrograms (Fig. 4), two main groups can be observed for both transplanted and *in situ* samples: (1) Fe, Al; (2) Zn, Cr, Pb, Cu. Within the second group, Cr and Pb eventually form a subgroup, but this occurs at a very low distance, indicating that this subgroup is not very robust. These same groupings have been obtained by other studies (Bozkurt, 2017; Brunialti & Frati, 2007; Rangel-Osornio et al., 2022).

The relationship between elements is used to identify groups with similar pattern and origin (Brunialti & Frati, 2007; Giordani et al., 2019). In this study, one of the clearest groups is Fe-Al. Loppi et al. (2000) also found this similar relationship between Fe and Al concentration. These elements are the main components of the Earth's crust, and their accumulation could be explained by the deposition of soil particles on the lichen surface (Loppi & Paoli, 2015). Once deposited on the lichen surface, Al and Fe accumulate by particle entrapment. These oxides of lithogenic elements are relatively insoluble in atmospheric particles and do not accumulate substantially through water-solubility processes. In fact, the presence of metals in particulate form explains the substantial accumulation of toxic elements in lichens in polluted environments (Brunialti & Frati, 2007).

All the elements in the second group (Zn, Cr, Pb, Cu) appear to originate from anthropogenic sources, as their presence is linked to activities such as combustion or various industrial environments (Bozkurt, 2017).

**Fig. 5:**
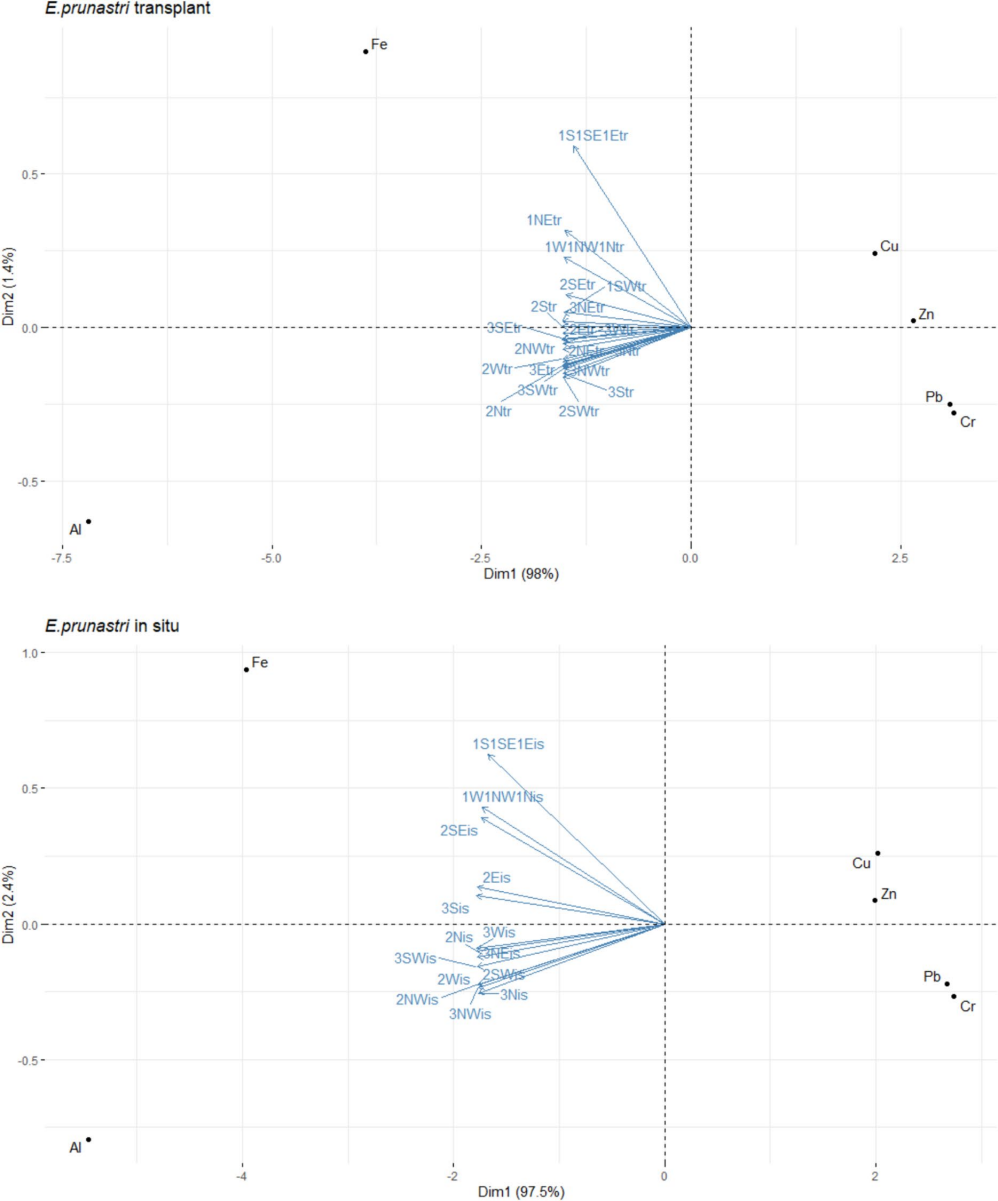
PCA showing the distribution and arrangement of the elements analysed. Transplant samples (up) and *in situ* samples (down)

PCA of transplanted and *in situ* samples (Fig. 5) revealed a clear separation between lithogenic elements (Fe, Al) and anthropogenic trace metals (Cu, Zn, Pb, Cr) along PC1, which accounted for 98% of the total variance, indicating a dominant gradient from natural background inputs to industrial contamination. The clustering of Cu, Zn, Pb, and Cr points to a common emission source, likely related to the recycling industry, while Fe and Al reflect soil-derived inputs.

### Total contamination and toxic trace metals spatial pattern

**Fig. 6:**
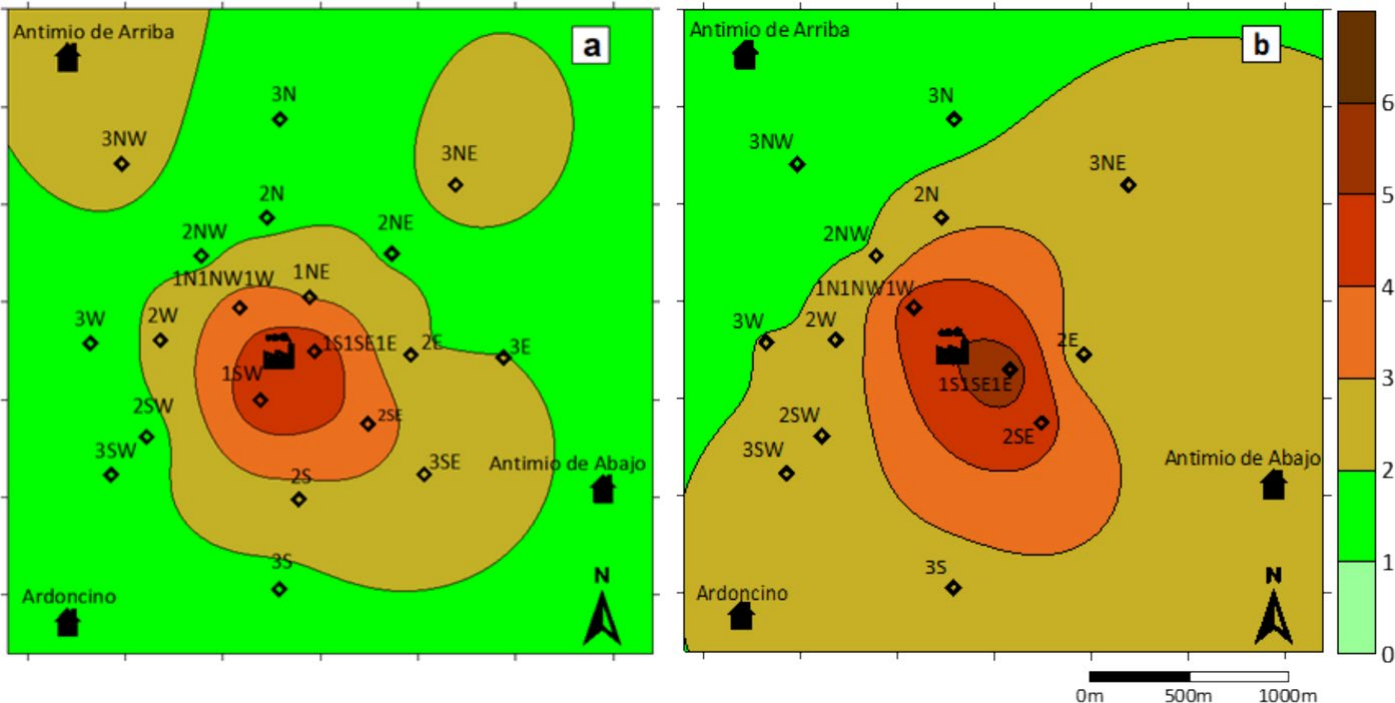
Spatial distribution map of the total contamination. The trace elements are indicated with values from 0 to 6. **a**: transplanted. **b**: *in situ* samples

Trace metals are classified as priority pollutants by the United States Environmental Protection Agency (Singh & Prasad, 2015). Figure 6 presents two maps of total contamination, showing that the highest contamination levels are concentrated in the vicinity of the recycling industry. Consistent with the individual dispersion patterns of each element, *in situ* samples indicate a broader spatial distribution of contaminants, likely due to their longer exposure time. In addition, a general dispersion pattern towards the NW–SE is observed, suggesting the transport of contaminants towards Antimio de Abajo village.

**Fig. 7:**
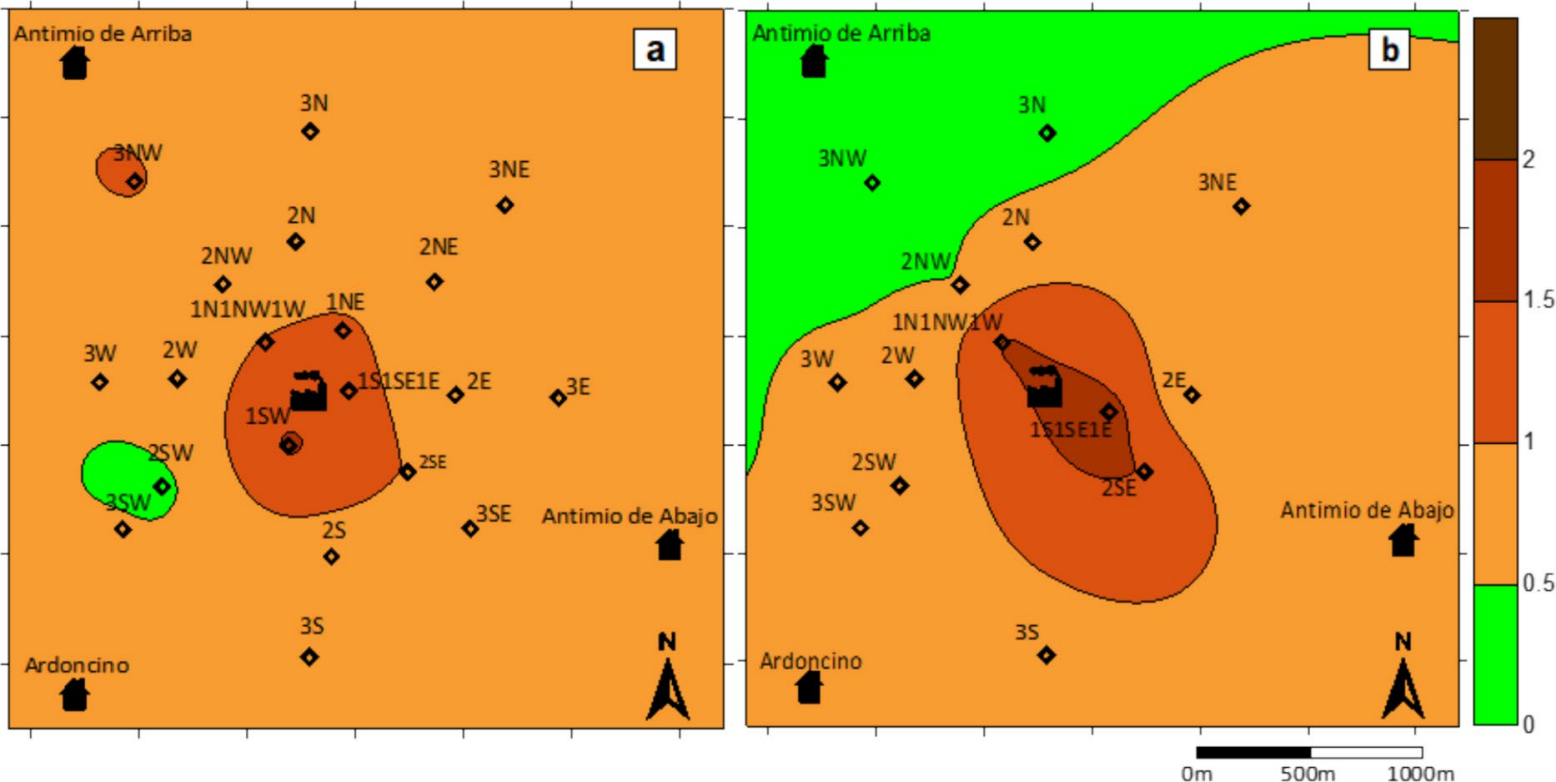
Spatial distribution map of elements toxic to humans (Cr and Pb). The two trace elements are indicated with values from 0 to 2. **a**: transplanted samples. **b**: *in situ* samples

Among the six elements analysed, Pb and Cr present a well-documented toxicity to plants, animals, and humans (Nordberg et al., 2015). Pb is ranked as one of the most hazardous substances by the United States Agency for Toxic Substances and Disease Registry (Singh & Prasad, 2015) and poses significant risks, especially to children, due to its effects on neurological development, behaviour, and cognitive function (EPA, 2025). Similarly, Cr can impact immunological, renal, and respiratory systems, and chromium VI compounds are classified as carcinogenic to humans (ATSDR, 2021). Figure 7 shows that both elements are concentrated near the industrial source and decrease with distance, following a NW–SE dispersion pattern. These results indicate that, among the three localities studied, Antimio de Abajo is the most affected by industrial contamination based on lichen accumulation patterns. However, further investigation is needed to assess whether these elements may accumulate in surrounding crops, predominantly cereals, and potentially enter the food chain.

## Conclusions

This study establishes the atmospheric trace metal distribution patterns in the surroundings of a recycling industry in León (NW Spain) using the lichen *Evernia prunastri* as a bioaccumulator, both *in situ* and in transplant experiments, and identifies Cu and Zn as the most relevant potential pollutants. The use of lichens as bioaccumulators of atmospheric trace elements remains limited in Spain, and this study provides novel evidence of their effectiveness in detecting spatial pollution patterns associated with recycling activities.

Enrichment factor analysis revealed a clear anthropogenic influence on lichen chemistry for Cu and Zn, and to a lesser extent Pb, confirming their atmophilic pattern and susceptibility to long-range atmospheric transport. In contrast, Al, Cr, and Fe were primarily associated with soil-derived inputs. Spatial distribution patterns and concentration–distance analyses further supported the industrial origin of Cu, Zn, and Pb emissions, as well as the influence of prevailing winds, particularly for Cr, Fe, and Zn in *in situ* samples. Risk maps enabled the identification of affected areas, although current contaminant levels do not pose an immediate risk to human health.

The enrichment factor proved to be a key tool for data interpretation and is therefore strongly recommended for future bioaccumulation studies. *Evernia prunastri* demonstrated excellent performance as a bioaccumulator under both transplant and *in situ* conditions, supporting its suitability for atmospheric biomonitoring. However, due to economic constraints, only one composite sample per site was analysed, and replication was not possible, which may have reduced statistical robustness. Nevertheless, the combined use of transplanted and *in situ* lichens allowed methodological validation, demonstrating comparable effectiveness and enabling meaningful comparisons with previous studies.

These findings highlight the value of lichen biomonitoring as a reliable and cost-effective tool for assessing atmospheric pollution associated with industrial activities. Further research should evaluate the potential transfer of these elements to nearby crops and their possible entry into the food chain, as well as reassess trace elements bioaccumulation in the same area through long-term monitoring incorporating replicate sampling to improve statistical robustness. Continuous air quality monitoring is therefore strongly advised.

## Data Availability

The datasets generated during and/or analysed during the current study are available from the corresponding author on reasonable request.
